# Techno-Economic Assessment of Bio-Syngas Production for Methanol Synthesis: A Focus on the Water–Gas Shift and Carbon Capture Sections

**DOI:** 10.3390/bioengineering7030070

**Published:** 2020-07-04

**Authors:** Aristide Giuliano, Cesare Freda, Enrico Catizzone

**Affiliations:** ENEA–Italian Agency for New Technologies, Energy and Sustainable Economic Development, Department of Energetic Technologies, Trisaia Research Centre, I-75026 Rotondella, Italy; cesare.freda@enea.it (C.F.); enrico.catizzone@enea.it (E.C.)

**Keywords:** biomass gasification, bio-methanol, process simulation, carbon capture, economic analysis

## Abstract

The biomass-to-methanol process may play an important role in introducing renewables in the industry chain for chemical and fuel production. Gasification is a thermochemical process to produce syngas from biomass, but additional steps are requested to obtain a syngas composition suitable for methanol synthesis. The aim of this work is to perform a computer-aided process simulation to produce methanol starting from a syngas produced by oxygen–steam biomass gasification, whose details are reported in the literature. Syngas from biomass gasification was compressed to 80 bar, which may be considered an optimal pressure for methanol synthesis. The simulation was mainly focused on the water–gas shift/carbon capture sections requested to obtain a syngas with a (*H*_2_ – *CO*_2_)/(*CO* + *CO*_2_) molar ratio of about 2, which is optimal for methanol synthesis. Both capital and operating costs were calculated as a function of the *CO* conversion in the water–gas shift (WGS) step and *CO*_2_ absorption level in the carbon capture (CC) unit (by Selexol^®^ process). The obtained results show the optimal *CO* conversion is 40% with *CO*_2_ capture from the syngas equal to 95%. The effect of the WGS conversion level on methanol production cost was also assessed. For the optimal case, a methanol production cost equal to 0.540 €/kg was calculated.

## 1. Introduction

Gasification is a thermochemical process for the conversion of a solid organic material, typically coal or biomass, into syngas. From a chemical point of view, gasification occurs due to the partial oxidation of a solid material at temperatures of 700–900 °C by means of a gasifying-oxidant agent, such as air, oxygen, steam or carbon dioxide. Gasification involves three main steps, namely, (i) solid drying; (ii) solid pyrolysis with formation of gases, volatile compounds and char; and (iii) secondary reactions, which convert the pyrolysis gas to syngas. Globally, gasification is an endothermic process; therefore, the system requires an energy input. The produced syngas is a mixture of incondensable gas at room temperature and atmospheric pressure that usually are carbon monoxide, hydrogen, carbon dioxide, methane and light hydrocarbons. In the case air is used as the gasifying agent, nitrogen is also present. Syngas from biomass has gathered huge attention in the last decades because it can be considered as a renewable source of energy, as the carbon dioxide released from syngas utilization is balanced by the carbon dioxide sequestration by plants during photosynthesis. For decades syngas has been proposed as fuel for cooking, lighting or for car engines. More recently, it has been used for combined heat and power production in internal combustion engines (ICEs). For this application, air is usually used as the gasifying agent due to its low cost. A syngas with a heating value of 4–5 MJ/Nm^3^_dry_ is usually obtained, which must be carefully cleaned of particulates and tar below 0.05 g/Nm^3^ and 0.1 g/Nm^3^, respectively, before being used in an engine [1]. Worldwide, extensive expertise has been acquired with a gasifier coupled with an ICE with power from tens of kWe to a few MWe [2,3,4,5]. Beyond the power production, the production of strategic green chemicals (e.g., methane, methanol and DME) from syngas is attracting the attention of the scientific community because it reduces the greenhouse gas emission and it partially preserves the chemical energy of syngas.

In particular, biomass gasification may be a sustainable way to produce methanol, which may be considered a strategy to introduce green energy in the industrial chain for chemicals and fuels. In fact, methanol is considered one of the most valuable green energy carriers of the future, since it may replace fossil sources (e.g., coal, natural gas or crude oil) for producing chemicals and fuels, [6,7,8]. Currently, several industrial plants produce dimethyl ether, olefins or gasoline starting from methanol [9,10,11,12]. Methanol is actually produced from syngas, usually obtained by means of steam reforming of natural gas. The syngas-to-methanol reaction is conventionally performed in a fixed-bed tubular reactor operating at 60–100 bar and 250–280 °C over a Cu–ZnO-based catalyst. In order to use conventional technologies, biomass-derived syngas needs to be treated and upgraded to obtain a syngas with the proper characteristics for methanol synthesis. For instance, tar removal is of paramount importance to prevent the catalyst’s deactivation. Both chemical and thermal techniques may be used for tar removal, although catalytic conversion seems to be the most effective way to reduce the tar content and convert them into syngas or light hydrocarbons [13,14]. An important aspect to consider is the (*H*_2_ – *CO*_2_)/(*CO* + *CO*_2_) ratio. In fact, with conventional technologies for methanol synthesis, the optimal (*H*_2_ – *CO*_2_)/(*CO* + *CO*_2_) molar ratio should be equal to 2 [15].

Unfortunately, syngas is far from having the above-written ratio, mainly because of elemental composition of the biomass is quite deficient in hydrogen compared to carbon. Hydrogen is only 6–7 wt% while carbon is about 47–49 wt% of the dry, ash-free biomass. This drawback could be theoretically overcome by a proper choice of the gasifying agent and reactor design. For instance, the utilization of steam as a gasifying agent improves hydrogen yield by means the three following reactions:

(1) $$C+H_{2}O=CO+H_{2}\;\;\;\;\;\;\;\;\;\;\Delta H{}^{\circ}\left(298\;K\right)=131\frac{\mathrm{kJ}}{\mathrm{mol}}$$

(2) $$CH_{4}+H_{2}O=3H_{2}+CO\;\;\;\;\;\Delta H{}^{\circ}\left(298\;K\right)=206\frac{\mathrm{kJ}}{\mathrm{moll}}$$

(3) $$CO+H_{2}O=CO_{2}+H_{2}\;\;\;\;\;\;\Delta H{}^{\circ}\left(298\;K\right)=-41\frac{\mathrm{kJ}}{\mathrm{mol}}$$

The reactants of Reactions (1)–(3), namely *C*, *CH*_4_ and *CO*, are during the biomass pyrolysis step. The involved reactions, as well as the Boudouard reaction, methanation, drying and pyrolysis, are globally endothermic; therefore, heat supply is necessary to sustain the gasification process at 800–900 °C. For instance, the reactor may be heated by an external thermal source. In this case, the performances of the heat exchange and the global efficiency of the process should be considered.

McCaffrey et al. performed gasification tests of almond shells on a bench scale, electrically heated, bubbling fluidized bed gasifier by using steam as the gasifying agent. They detected interesting results in terms of the syngas composition: 35–40 vol% hydrogen, 18–21 vol% carbon monoxide, 16–18 vol% carbon dioxide, 17–21 vol% nitrogen and 5–6 vol% methane [16]. Nevertheless, with the goal for scaling-up the process from a bench to industrial scale, the heat supply for the endothermic gasification reactions should be provided in a simpler and efficient way. In this regard, the injection inside of the reactor of an oxidant agent, such as air or oxygen, is a prosecutable way. In fact, Campoy et al. gasified biomass by means of an air–steam mixture in a fluidized bed gasifier under simulated autothermal and adiabatic conditions, with the aim of reproducing the behavior of a full-scale fluidized bed gasifier [17]. They detected a syngas with the following composition: 12–16 vol% hydrogen, 12–15 vol% carbon monoxide, 16–19 vol% carbon dioxide, about 5 vol% methane and about 50 vol% nitrogen. They also showed that a proper selection of operating conditions makes it possible to increase the gasification efficiency from 40% to 60%. On the other hand, the main drawback of the air–steam gasification is related to the large dilution in nitrogen, with all the negative consequences on the thermodynamic and kinetics of the methanol synthesis, which is favored at high partial pressures of the reactants. Therefore, the utilization of pure oxygen instead of air has undoubted advantages concerning the production of a syngas for methanol synthesis. On the other hand, an additional cost is associated with pure oxygen production, although it may have some advantages in terms of capital investment costs linked to the reduced size of the reactor and related equipment because of the reduced volumetric flows. Studies on the relatively low-cost technology for oxygen production strengthen the feasibility of syngas production from oxygen–steam gasification [18,19]. Meng et al. gasified different biomass in a 100 kW_th_ steam–oxygen-blown circulating fluidized bed gasifier (CFB). They observed that the product gas composition obtained from willow over the temperature range from 800 to 820 °C consisted of a syngas with a high *H*_2_ concentration (29 vol%), and with *CO* (22 vol%), *CO*_2_ (38 vol%), *CH*_4_ (7 vol%) and C_2_-C_3_ (3 vol%) [20]. Barisano et al. gasified almond shells in a 1 MW_th_ pilot plant based on a bubbling fluidized bed gasifier with internal recirculation by using a mixture of enriched air–steam and oxygen–steam. Obviously, the best results in term of syngas composition for methanol production were obtained in the case of pure oxygen–steam gasification at process temperatures of 820–830 °C, with 30–33 vol% hydrogen, 28–32 vol% carbon monoxide, 22–27 vol% carbon dioxide, 9–11 vol% methane, 1–2 light hydrocarbons and 2–5 vol% nitrogen [21]. An alternative way for supplying thermal energy for endothermic steam gasification was developed at TUW: it consists of the utilization of the bed material as a heat carrier in a dual fluidized bed. The obtained syngas showed the following composition: *H*_2_ (36–42 vol%), with *CO* (19–24 vol%), *CO*_2_ (20–25 vol%), *CH*_4_ (9–12 vol%) and C2-C3 (3–4 vol%) [22]. From this short review about biomass steam gasification, it can be argued that, despite of these efforts, the syngas’s (*H*_2_ – *CO*_2_)/(*CO* + *CO*_2_) ratio is still far from 2; therefore, additional steps need to be implemented in order to both increase the *H*_2_ content and reduce the *CO*_2_ level, although several efforts have been done in last decades aimed to develop catalysts for *CO*_2_-rich syngas conversion towards methanol or methanol-derivates [23,24,25,26,27]. Feng et al. analyzed the production of methanol from biomass gasification by simulating a plant mainly consisting of a biomass gasifier, steam reformer and methanol synthesis reactor [28]. The authors adopted a pressure swing adsorption (PSA) unit to separate hydrogen from the unreacted gases and recycling it to the reactor inlet. With this approach, the (*H*_2_ – *CO*_2_)/(*CO* + *CO*_2_) molar ratio in the methanol reactor inlet was increased up to 0.7, which is still far lower than the ideal value. PSA was also adopted in the study of Puig-Gamero in order to adjust the composition of syngas coming from a gasification unit [29].

A water–gas shift reaction (WGS, *CO* + *H*_2_O = *CO*_2_ + *H*_2_) represents the most used method to increase the hydrogen content in syngas. WGS is normally used after steam reforming or partial oxidation of natural gas or coal to produce hydrogen-rich syngas. The WGS is an exothermic reversible reaction. Due to the thermodynamics constraint, WGS is normally performed in two separated reactors: a high temperature shift reactor (HTS) and a low temperature shift reactor (LTS). Following this approach, in the first reactor kinetics is favored due to the high temperature, while thermodynamics is favored in the low temperature reactor, allowing obtaining high *H*_2_ yield. HTS is normally performed up between 310 °C and 450 °C and 25–35 bar by using an iron/chromium catalytic system, where iron acts as active sites while the presence of chromium reduces the iron sintering [30]. The LTS step is usually carried out between 200 °C and 240 °C with a copper/zinc/alumina catalytic system able to convert the residual *CO* in the case pure hydrogen is desired. In order to obtain a syngas with a composition suitable for methanol synthesis, the WGS section needs to be integrated with a *CO*_2_ removal section. In this regard, absorption is the most used technology to remove *CO*_2_ from syngas streams. Ju et al. adopted a WGS downstream of the biomass gasification unit in order to increase the hydrogen content. In the WGS unit, the *CO* conversion was set at 70%, and the *CO*_2_ was removed by the Rectisol process [31]. Robinson et al. used the WGS coupled with Selexol, in order to produce pure hydrogen from syngas derived from a gasification unit. The obtained hydrogen-rich gas was mixed with the raw syngas to obtain a suitable composition for methanol synthesis. In this study, *CO* was almost totally converted in the WGS step, and about 90% of the *CO*_2_ was removed in the Selexol unit [32]. The aim of this work is to study in more detail, in terms of economic analysis, the WGS and carbon capture section (CCS). In particular, capital costs and operating costs were assessed as a function of the WGS conversion level, taking as the target a syngas suitable for methanol synthesis. An optimal WGS conversion/CCS configuration is suggested on the basis of the obtained results.

## 2. Materials and Methods

### 2.1. R-Ratio and WGS Conversion/Carbon Capture Relationship

As discussed in the introduction, the syngas produced by biomass gasification needs to be upgraded in order to obtain a (*H*_2_ – *CO*_2_)/(*CO* + *CO*_2_) molar ratio (here named R-ratio) equal to 2, optimal for methanol synthesis [33]. The following strategies can be used to adjust the R-ratio to the desired value:

(A) add pure *H*_2_ [34];
(B) remove *CO*_2_ [32].

The A strategy has an impact on the numerator of the R-ratio, and it is possible to obtain an R-ratio = 2 by adding a large quantity of pure hydrogen, as may be observed from the following equation:

(4) $$\frac{H_{2,IN}-CO_{2,IN}}{CO_{IN}+CO_{2,IN}}\to \frac{H_{2,In}+H_{2,PURE}-CO_{2,IN}}{CO_{IN}+CO_{2,IN}}$$

where *H*_2,*IN*_ is the inlet hydrogen molar flowrate, *CO*_2,*IN*_ is the inlet carbon dioxide molar flowrate, *CO_IN_* is the inlet carbon monoxide molar flowrate, and *H*_2,*PURE*_ is the inlet pure hydrogen molar flowrate. The main hurdle of this strategy is related to the production of pure hydrogen in a sustainable way. In fact, traditional processes for hydrogen production are based on fossil sources (e.g., coal, oil and natural gas) with a high *CO*_2_ emission, even though at a relatively low cost [35]. On the contrary, renewable electricity sources (e.g., wind) may be used to produce hydrogen with a low carbon footprint via water electrolysis [34]. Actually, the addition of pure hydrogen is clearly an expensive strategy, even though water electrolysis with atmospheric carbon dioxide capture is an attractive route to produce methanol [36].

The B strategy can be applied by means of different process schemes. In particular, *CO*_2_ removal can be simple or difficult (cheap or expensive), depending on whether the *CO*_2_ molar fraction (and partial pressure) is low or high, respectively [37].

The *CO*_2_ removal from clean syngas can be useless if the molar fraction of *H*_2_ and *CO* are not appropriate for methanol synthesis. For example, depending on the syngas quality, a 100% *CO*_2_ capture may produce an R-ratio (*H*_2_/*CO*) lower than 2, and thus not suitable for methanol synthesis.

On the other hand, if the *CO*_2_ molar fraction is too low, it is very difficult to achieve high *CO*_2_ capture in order to obtain an optimal R-ratio [38]. Therefore, the viability of the carbon capture section strongly depends on the syngas composition.

As mentioned in the introduction part, *CO* may be partially converted to hydrogen by the water–gas shift reaction, which would lead to an increase in both hydrogen and *CO*_2_ content. Nevertheless, by WGS only it is not possible to change the R-value, as perhaps easily demonstrated:

(5) $$\frac{H_{2,In}-CO_{2,IN}}{CO_{IN}+CO_{2,IN}}\to \frac{H_{2,In}+CO_{IN}x_{WGS}-\left(CO_{2,IN}+CO_{IN}x_{WGS}\right)}{CO_{IN}-CO_{IN}x_{WGS}+CO_{2,IN}+CO_{IN}x_{WGS}}=\frac{H_{2,In}-CO_{2,IN}}{CO_{IN}+CO_{2,IN}}\;$$

where *x_WGS_* is the *CO* conversion in the WGS section. Nevertheless, in this way, the *H*_2_ molar ratio increases as well as the *CO*_2_ molar ratio, making the *CO*_2_ removal simpler [39]. In this study, we used a syngas with a composition as reported in the paper of Barisano et al. [21]. Briefly, the authors reported a study about gasification of almond shells with a 1000 kWh pilot-scale, internally circulating, bubbling fluidized bed reactor, by using a steam/O_2_ mixture as the gasification agent. Both char and tar were abated with a ceramic filter and wet scrubber, respectively. The gas stream from the gasification unit is assumed to be cleaned from residual tars and sulfur compounds, obtaining a mixture with the average composition reported in Table 1.

As may be noted, *CO*_2_ removal is useless without a WGS step, due to the relatively low hydrogen content in the syngas. In particular, the minimum WGS conversion (corresponding to a 100% *CO*_2_ capture rate) is equal to about 33%, obtaining an R = 2:

(6) $$\frac{H_{2,IN}+CO_{IN}x_{WGS}-\left(CO_{2,IN}+CO_{IN}x_{WGS}\right)\left(1-1\right)}{CO_{IN}-CO_{IN}x_{WGS}+\left(CO_{2,IN}+CO_{IN}x_{WGS}\right)\left(1-1\right)}=2\to \;\frac{30+30x_{WGS}}{30-30x_{WGS}}=2\to \;x_{WGS}=0.33\;$$

Similarly, it is possible to individuate the minimum *CO*_2_ capture rate (corresponding to a 100% WGS conversion) equal to 64%:

(7) $$\frac{H_{2,IN}+CO_{IN}-\left(CO_{2,IN}+CO_{IN}\right)\left(1-\alpha_{CO2}\right)}{CO_{IN}-CO_{IN}+\left(CO_{2,IN}+CO_{IN}\right)\left(1-\alpha_{CO2}\right)}=2\to \;\frac{60-\left(55\right)\left(1-\alpha_{CO2}\right)}{\left(55\right)\left(1-\alpha_{CO2}\right)}=2\to \;\alpha_{CO2}=0.64$$

Furthermore, the *CO*_2_ capture rate (*α_CO2_*) as a function of WGS conversion *x_WGS_* may be calculated as follows:

(8) $$\alpha_{CO2}=\frac{2\;CO_{IN}-H2_{IN}+3\;CO_{2,IN}}{3\;\left(CO_{2,IN}+CO_{IN}\;x_{WGS}\right)}$$

**Figure 1:**
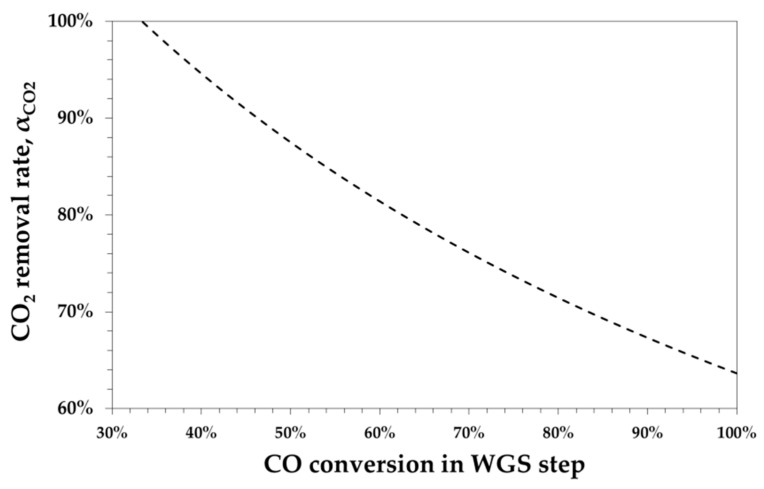
Calculated *CO*_2_ removal rate as a function of *CO* conversion in the water–gas shift (WGS) to obtain an R = 2.

Figure 1 shows that *α_CO2_* decreases with the WGS conversion.

### 2.2. Flowsheet Description

**Figure 2:**
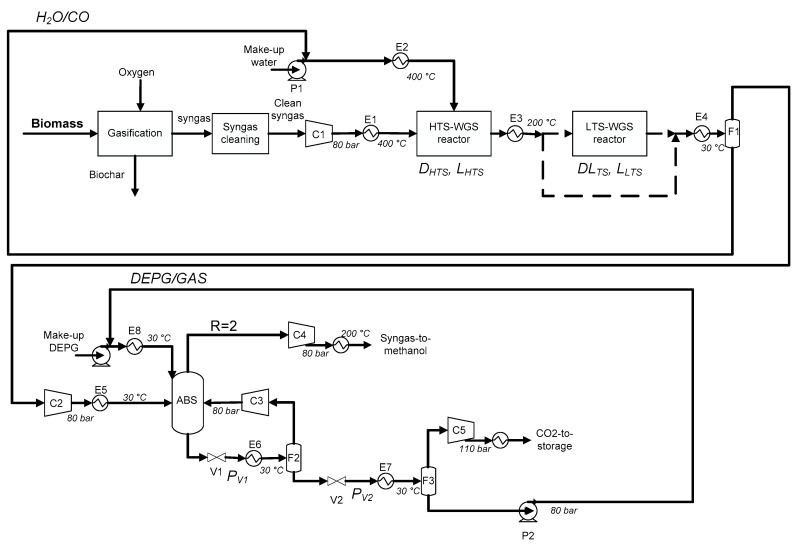
The syngas-to-methanol production flowsheet considered in this work.

Figure 2 shows the process flowsheet (drawn using Microsoft Power Point software) considered in this work based on two consolidate technologies: two-step WGS and *CO*_2_ absorption by the Selexol^®^ process.

After the syngas purification process, the syngas is compressed (C1) at the methanol synthesis pressure (here 80 bar). The heat exchanger E1 heats the compressed syngas for the HTS-WGS at 400 °C. Water is vaporized and superheated to 400 °C by E2. As mentioned in the introduction part, in the HTS-WGS reactor, Fe_2_O_4_/Cr_2_O_3_ is usually used as the catalyst [40]. WGS is an exothermic reaction, so in the adiabatic reactor the increase in temperature causes a decrease in the reaction equilibrium conversion [41]. Moreover, the higher the inlet temperature is, the lower will be the equilibrium conversion. So, if a high CO conversion is requested, LTS-WGS may be considered as well, in order to promote a reaction from a thermodynamics point of view. Catalysts used for this step is Cu–ZnO–Al_2_O_3_ and the temperature is set to 200 °C [42]. The heat exchanger E4 allows for the condensation of the residual water, which is recycled back to E2. *H*_2_/*CO*_2_-rich gas is then sent to a small compressor to reestablish the pressure at 80 bar due to the pressure drop in the WGS reactors. The absorption column ABS works at 30 °C in order to promote the absorption of *CO*_2_ in the solvent. Liquid outlet stream from the bottom of the column (rich in *CO*_2_) is expanded in order to recover the other volatile compounds (*H*_2_ and CO). A flash (F2) at 30 °C recycles these gases to the column. The DEPG solvent and *CO*_2_ are then expanded again by the V2 valve and the F3 flash allows the recovery of high pure *CO*_2_ (higher than 99% of purity) and regeneration of the solvent. Pure *CO*_2_ is finally compressed until 110 bar for the storage [43]. Regenerated DEPG is compressed again by the P2 pump and recycled. A gaseous *H*_2_-rich stream from the top of the ABS is compressed, if necessary, and heated to 200 °C, to be recycled to the methanol synthesis reactor.

A detailed list of the main items considered in this work with the type of auxiliary stream is reported in Table 2.

The main equipment in terms of utility consumption is further detailed here:

- C1 is the main compressor of the process, because the clean biomass-derived syngas is considered at 1 bar.
- E2 is the main heat exchanger of the process, because it vaporizes water at high pressure (80 bar); at this pressure the vaporization temperature of water is about 295 °C, so a boiler (fueled with natural gas) is necessary for both vaporization and superheating. Hence, the *H*_2_*O*/*CO* ratio for the WGS sets the thermal power (and the natural gas demand) of E2.
- C3 is the compressor of the gaseous stream recycled to the ABS column and the electricity required depends on the pressure drop at the throttling valve necessary to release volatile compounds (*H*_2_ and *CO*), leaving *CO*_2_ into the solvent.
- C5 is considered to compress *CO*_2_ at 110 bar for storage [43].
- P2 is an energy-intensive pump, as a large amount of DEPG solvent is requested to make the *CO*_2_ capture possible.

Considering the general flowsheet of Figure 2, several process parameters can be modified in order to obtain different *x_WGS_* and *α_CO2_* and, consequently, an R-ratio equal to 2. The L/D ratio of the WGS reactors was set equal to 8.

Furthermore, Table 3 shows the process variables investigated in this work and their effect on the process.

One of the main process variables to increase the *CO* conversion in the WGS unit is the *H*_2_*O*/*CO* ratio. The WGS reaction is limited by thermodynamics, therefore theoretical conversion of *CO* may be increased by increasing the *H*_2_*O*/*CO* ratio. To have a good reactor set up, a minimum *H*_2_*O*/*CO* ratio of 1 is necessary. With the increase in the *H*_2_*O*/*CO* ratio, a higher *CO* conversion can be obtained, but the heating power of E2 can become too high to justify higher conversions. In order to limit the pressure drop, parallel adiabatic tubular reactors with the same length and diameter were considered. So, a higher number of tubes must be used in order to save electricity at C2.

With the increase in the number of tubes, a higher volume is obtained and, consequently, a higher conversion, with an acceptable pressure drop (less than 2 bar) [44].

For the carbon capture section, both the DEPG/GAS ratio and expansion pressure of V1 and V2 have to be set in order to have an R-ratio equal to 2, and low values of solvent quantity and electricity of the P2, C3 and C5 items

In particular, a higher DEPG/GAS ratio correspond to a higher absorbed quantity of *CO*_2_, *H*_2_ and *CO*. Consequently, lower V1 pressures may be set to recover *H*_2_ and *CO*, and therefore a higher electricity demand for both the C3 and P2 items were calculated. A lower DEPG/GAS ratio correspond to a lower absorbed quantity of *CO*_2_, *H*_2_ and *CO*, which may be insufficient for obtaining the *α_CO2_* required for the correspondent *x_WGS_*, and the V2 pressures have to be then set to a lower value in order to completely regenerate the solvent, then requiring a higher C5 electricity demand.

### 2.3. Process Simulation Description

A clean syngas with a composition as in Table 1 was fed to the process simulation scheme as reported in Figure 2. Pumps and compressors were considered adiabatic devices with an efficiency equal to 0.85 and 0.75, respectively. Hot streams with a temperature in the range 100–500 °C were coupled with cold streams with a temperature in the range 0–200 °C, for heat integration purposes. Cold streams at temperatures higher than 200 °C were considered supplied by a boiler fueled with natural gas.

HTS-WGS kinetics from Hla et al. (2009) [40] and LTS-WGS kinetics from Choi et al. (2003) [42] were adopted for the simulation. Both the WGS reactors were considered adiabatic and pressure drop was calculated by the Ergun relationship [43]. Thermodynamic models used for the simulation were the NRTL model for the estimation of activity coefficients for the liquid phase and the Soave–Redlich–Kwong equation for the gas phase [45]. An equilibrium absorption column was used for ABS.

In order to fix the *x_WGS_*-*α_CO2_* working points, a sensitivity analysis was carried out by varying the process variables reported in Table 3. Considering also the impact of the process variables on the process economics, from the sensitivity analysis were selected the WGS reactor sizes with the lowest lengths (and the lowest pressure drops), the lowest *H*_2_*O*/*CO* ratio, the lowest DEPG/GAS ratio and the maximum V1 and V2 pressures.

### 2.4. Economic Assessment Description

For the economic assessment of the whole process, both CAPEX and OPEX costs were evaluated.

In particular, the biomass supply chain and pure oxygen costs, natural gas cost, electricity cost, maintenance and labor costs were considered as the operating costs (OPEX). The economic data used in the analysis are reported in Table 4. A biomass supply chain cost equal to 40 €/t was considered from Menin et al. (2020) [46] for a plant size equal to 147,000 t/y (18.6 t/h) of biomass fed to the gasifier, producing 861 kmol/h of syngas. Maintenance and labor costs were associated to the capital costs and estimated as 10% of the annual total capital costs.

The capital costs considered were the following:

- Gasifier + gas cleaning system [52].
- All pumps and compressors for the WGS and CC sections [49].
- WGS reactors, considering shell and catalysts separately [49].
- ABS column, considering shell and packing separately [49].

For all the equipment, both the scale economies, temperature and pressure ranges were considered as follows:

(9) $$CC_{j}=CC_{0,j}{\left(\frac{Q_{j}}{Q_{0,j}}\right)}^{m_{j}}F_{i}F_{t}F_{p}$$

where *CC_j_* is the capital cost of the j-equipment, *CC*_0,*j*_ is the capital cost of the j-equipment for the size *Q*_0,*j*_, *Q*_0,*j*_ is the base size for the j-equipment, and *F_t_* and *F_p_* are the temperature level factor and the pressure level factor, respectively. *F_i_* is the installation factor reported in the references where cost data are reported [49,52].

A 4-year catalyst charge was set in the cash flow analysis in order to calculate the production cost of the methanol, and the global methanol yield from the syngas with an R-ratio = 2 was set to 80% [53]. The capital costs for the methanol synthesis unit were taken from Reference [54].

The NPV (net present value) was set equal to 0, and the corresponding value of the selling price of the produced methanol was then calculated as follows:

(10) $$NPV=\overset{n}{\underset{i=0}{\sum }}\frac{CF_{i}}{{\left(1+r\right)}^{i}}=0$$

(11) $$CF_{i}=\left(P_{MeOH}F_{MeOH,i}-OC_{i}\right)\left(1-t\right)+WC_{i}+\frac{CC_{TOT}}{y_{D}}t-CC_{i}$$

where *CF_i_* are the cash flow of the year *i*, *r* is the discount rate, *n* is the final year (22), *P_MeOH_* is the production price of syngas-to-methanol, *F_MeOH,i_* is the molar flowrate of methanol for the year *i*, *OC_i_* are the operating costs for the year *i*, *t* is the tax ratio, *WC_i_* is the working capital, *CC_TOT_* are the total capital costs, and *y_D_* are the depreciation years.

The working capital was set as a negative value at the start-up year (*i* = 2), and as a positive value at the last year (*i* = 21). The construction of the plant was considered completed between years 0 and 1 (equal capita cost each year).

## 3. Results

### 3.1. Process Results

Table 5 shows the process simulation results in terms of the process variables values obtained from the simulation, sensitivity, economic analysis and variable optimization.

In particular, the minimum *CO* conversion in the WGS step was set to 36%, while the maximum one to 99%. By varying the HTS-WGS reactor length, the WGS conversion equaled 36%, or 40%, and the *H*_2_*O*/*CO* equaled 1; a one-step WGS was sufficient to reach the conversion desired. Furthermore, for the WGS conversion of 49% and 59%, only HTS was sufficient, but a *H*_2_*O*/CO equal to 2 was required, because the addition of the LTS reactor is not sufficient to reach the desired conversions.

By increasing the *CO* conversion from 36% to 59%, which correspond to a *CO*_2_ capture rate decrease from 98% to 82%, respectively, the DEPG/CAS molar ratio may be decreased from 1 to 0.7. V1 pressure varies between 19 and 25 bar; furthermore, the V pressure decreases as the *α_CO2_* value decreases. When the *CO*_2_ partial pressure in the syngas-fed inlet to the column is higher, the *CO*_2_ capture is favored, but the required *α_CO2_* is lower. Consequently, a higher flowrate of the gas recycled to the column is calculated, and a lower V1 pressure needs to be set. The optimal pressure of the V2 valve increases as the *CO*_2_ capture decreases, because a lower regeneration level of the solvent is requested, also in relationship with the DEPG/GAS ratio.

A *H*_2_*O*/*CO* molar ratio equal to 2 was sufficient for a *CO* conversion in the WGS equal to 70% and 80%, while the *H*_2_*O*/*CO* molar ratio has to be increased to 3 for a conversion higher than 90%. In these cases, the LTS-WGS unit is necessary from a thermodynamics point of view, and then 70%, 80%, 90% and 99% *x_WGS_* may be obtained. The *CO* conversion reaction rate is slower in the LTS-WGS due to a lower reactant concentration and reaction temperature with respect to the HTS-WGS step.

In order to obtain a *CO*_2_ capture rate of 76%, 72%, 67% and 64% after the WGS units, which correspond to 70%, 80%, 90% and 99% of the *CO* conversion, respectively, a DEPG/GAS ratio was set equal to 0.5. A maximum V1 pressure of 58 bar (minimum compressor power demand of C3 compressor) was found for a *CO* conversion of 70%. This high pressure is due to the high concentration of *CO*_2_ in this stream. To obtain the regeneration of the solvent, the lowest value of the V2 pressure was necessary (0.7 bar). The decrease in the *α_CO2_* values cause a decrease in V1 pressure in order to recover a small quantity of *CO*_2_ solubilized in the column outlet stream for these cases.

**Figure 3:**
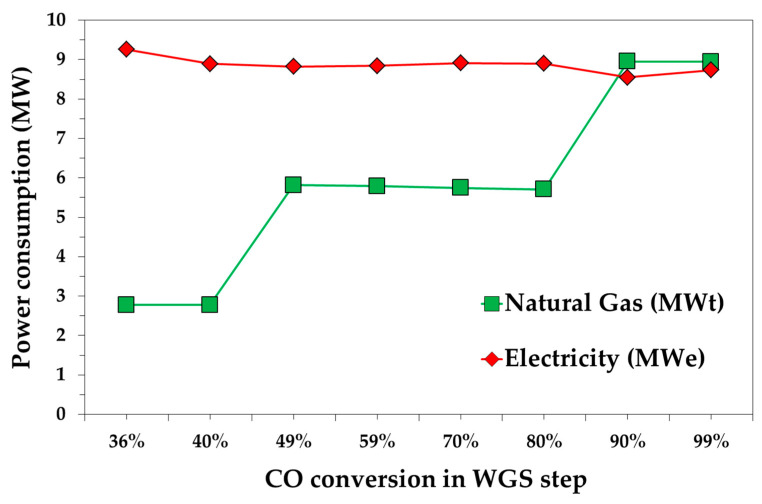
Natural gas and electricity required as function of *CO* conversion in the WGS step.

Figure 3 shows both the thermal and the electricity power demand as a function of *CO* conversion in the WGS steps. In particular, the natural gas demand obviously increases by increasing the *H*_2_*O*/*CO* ratio for the WGS reactor due to the higher production of steam. For an *x_WGS_* equal to 36% and 40%, which requires a *H*_2_*O*/*CO* ratio equal to 1, about 2.7 MWt of thermal energy is calculated. About a double value was calculated for a *CO* conversion in the range of 49–80%, which were obtained with a *H*_2_*O*/*CO* ratio equal to 2. A slighter effect of *CO* conversion on electricity power demand was calculated, as it varies between 8.6 and 9.3 MWe. A more careful analysis reveals that the minimum value was achieved at a 90% *CO* conversion. Electrical demand is mainly related to the DEPG recycle pump (P2) and *CO*_2_ compressor (C5) units. Both the power demands increase as the *CO* conversion decreases. For *x_WGS_* = 99%, the recovery compressor C3’s power is higher than *x_WGS_* = 70%, 80% and 90%, due to the lower V1 pressure.

**Figure 4:**
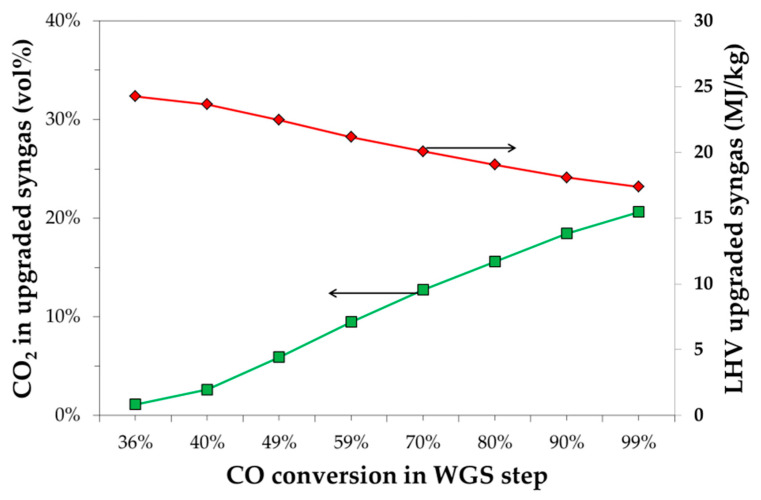
The effect of *CO* conversion in the WGS step on the *CO*_2_ content (left axes) and low heat value (right axes) of the post-CCS, upgraded syngas.

Figure 4 reports the characteristics of the upgraded syngas in terms of *CO*_2_ content and LHV. *CO*_2_ content increases by increasing the *CO* conversion, increasing from about 1% to about 21% by increasing the *CO* conversion from 36% to 99%, while the *CO*_2_ removal decreases from 98% to 64%. The *CO*_2_ molar fraction obviously has an impact on the lower heating value of the upgraded syngas that decreases from about 25 MJ/kg to about 18 MJ/kg, by increasing the *CO*_2_ content from 1% to 21%.

Methanol synthesis occurs by hydrogenation of the *CO* and *CO*_2_ compounds, so in the stream syngas-to-methanol, only *CO* and *CO*_2_ can be converted to methanol. The carbon loss due to the WGS/CCS unit was about 64%, regardless of the *CO* conversion in the WGS steps.

This result may be obtained by the mathematical relationships between the R-ratio, *x_WGS_* and *α_CO2_*. The captured carbon atoms were calculated as follows:

(12) $$y_{CO2}\left(CO_{2,IN}+CO_{IN}\;x_{WGS}\right)=\frac{2\;CO_{IN}-H2_{IN}+3\;CO_{2,IN}}{3\;\left(CO_{2,IN}+CO_{IN}\;x_{WGS}\right)}\left(CO_{2,IN}+CO_{IN}\;x_{WGS}\right)\;=\frac{2}{3}CO_{IN}-\frac{1}{3}H2_{IN}+CO_{2,IN}$$

So, both *x_WGS_* and *α_CO2_* do not affect the carbon capture level.

### 3.2. Economic Results

Four main sections were considered for the estimation of capital costs: (i) gasification and syngas cleaning; (ii) the water–gas shift; (iii) carbon capture and storage; and (iv) methanol synthesis.

**Figure 5:**
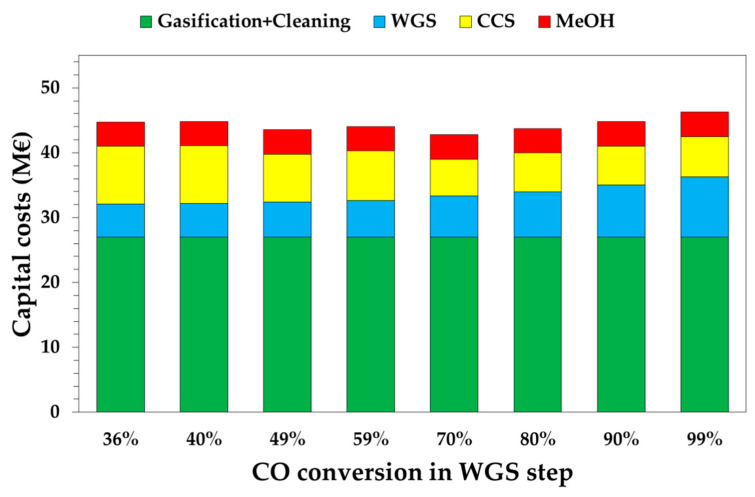
Capital costs as a function of WGS conversion. Gasification includes syngas cleaning.

Figure 5 shows the capital cost distribution as a function of the WGS conversion. Green bars represent the gasification and syngas cleaning section and it is equal to 27 M€, regardless of the WGS conversion level. On the contrary, WGS affects the capital costs of the WGS and CC sections, which have an opposite trend. To reach higher *CO* conversions, higher capital costs are estimated for the WGS section, while lower capital costs are estimated for the CC section; this thanks to higher partial pressures of the *CO*_2_ in the shifted syngas after favoring *CO*_2_ absorption during the Selexol^®^ process. For low WGS conversions (i.e., 36–59%), the capital cost of the WGS section is below 6 M€ due to the absence of the LTS-WGS reactor. The highest value of the CC section is calculated for a 99% WGS conversion, with a value of 9.2 M€. On the other hand, the CC section is below 7 M€ for *CO* conversions in the range 70–99%, with the lowest value for *CO* conversion equal to 70% (i.e., 5.7 M€). The lowest capital costs of the CC section is calculated for a conversion of 70%, due to (i) a lower DEPG flowrate in the column, which requires a smaller column diameter; (ii) a smaller size pump at P2; and (iii) a lower amount of solvent to buy for the start-up of the absorption unit.

Globally, the combination of these effects leads to estimate that the minimum value of the capital costs at the point where *x_WGS_* = 70% is 42.8 M€. The most expensive case is for *x_WGS_* = 99%, at 46.3 M€.

**Figure 6:**
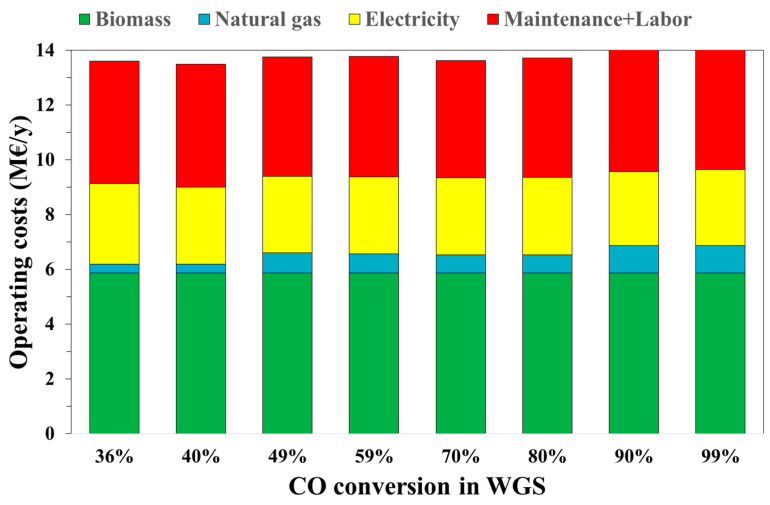
Operating costs varying with the WGS conversion.

Operating costs have a higher impact on the economic analysis. In fact, the lowest operating costs are for the case *x_WGS_* = 40% (13.1 M€/y) and the highest operating costs are for *x_WGS_* = 99%, at 13.8 M€/y, as shown in Figure 6.

Figure 6 shows the operating costs of the process as a function of the WGS conversion value. In particular, a constant cost is for the biomass supply (about 5.9 M€/y). The natural gas cost increases by increasing the *CO* conversion due to a higher *H*_2_*O*/*CO* ratio requested to obtain the desired conversion. This is the main effect of the *H*_2_*O*/*CO* ratio on the operating costs. A slighter effect is calculated in terms of electricity costs (2.7–2.9 M€/y). Maintenance and labor costs follow the same trend in capital costs since they were considered equal to 10% of the latter.

**Figure 7:**
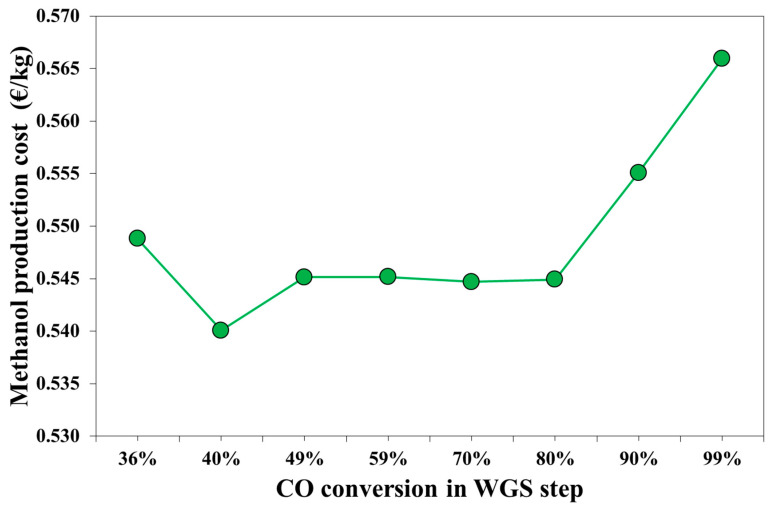
Cost of the upgraded syngas for methanol synthesis expressed in kmol of carbon atoms.

The effect of WGS conversion on methanol production cost is reported in Figure 7. The minimum production cost was calculated for a WGS conversion equal to 40%, with a value equal to 0.540 €/kg. The highest impact on the production cost were derived from the operating costs. Considering a 20-year plant life, the mean values of the annualized capital costs is equal to 2.2 M€/y, while the mean operating costs are about 13.4 M€/y. As shown in Figure 3, the natural gas demand increases with the *H*_2_*O*/*CO* ratio. The electricity demand for the case *x_WGS_* = 36% is 4% higher than electricity demand calculated for the optimal case, *x_WGS_* = 40%, due to the different electricity demanded by C5, which was caused by a lower V2 pressure. For a *CO* conversion between 49% and 80%, the methanol production cost is similar, which is about 0.545 €/kg, and depends on the natural gas demand.

For the WGS conversion of 90% and 99%, the natural gas cost is the main cause of higher production cost. For these cases, also the capital costs are higher than the case with a lower WGS conversion, due to the higher capital costs of both the WGS reactors (associated to *CO* conversion) and the ABS column due to a higher gas flowrate.

Obtained results may be affected by several variables, such as the syngas final pressure, which here was set to 80 bar. A higher pressure in the WGS section does not have an influence on the reaction equilibrium, but a higher partial pressure of the reactants would favor reaction kinetics, then requiring a smaller size of the reactors. Furthermore, the CC section is strongly affected by syngas pressure, because *CO*_2_ absorption would be more favored and, consequently, an optimal column size and solvent-to-gas ratio would be lower. On the other hand, the C1 compressor pressure also has an effect on the global economic analysis. In particular, in decreasing the C1 pressure, both the WGS reactor and CC section equipment size (column size, solvent flowrate) would be higher.

In the investigated process, since the optimal case was for a *CO* conversion equal to 40% and a *CO*_2_ capture equal to 95%, the optimal configuration was without an LTS-WGS reactor and both the *H*_2_*O*/*CO* and DEPG/GAS ratios equal to 1. In that case, operating costs were the lowest (13.1 M€/y), divided into 5.9 M€/y for biomass supply, 4.5 M€ for maintenance and labor, 2.8 M€/y for electricity and only 0.3 M€/y for the natural gas consumption. In particular, electricity consumption is required by the syngas compressor C1 (about 6.6 MWe), the *CO*_2_ compressor C5 (about 1.4 MWe) and by solvent pump P2 (about 0.66 MWe). Total capital costs were about 44.8 M€, as a sum of 27 M€ for the gasification system, 7.0 M€ for the compressors, 1.4 M€ for the pumps, 4.4 M€ for the Selexol^®^ system and 1.1 M€ for the WGS. Compressor C1 was the most expensive compressor with a cost of 4.1 M€.

**Figure 8:**
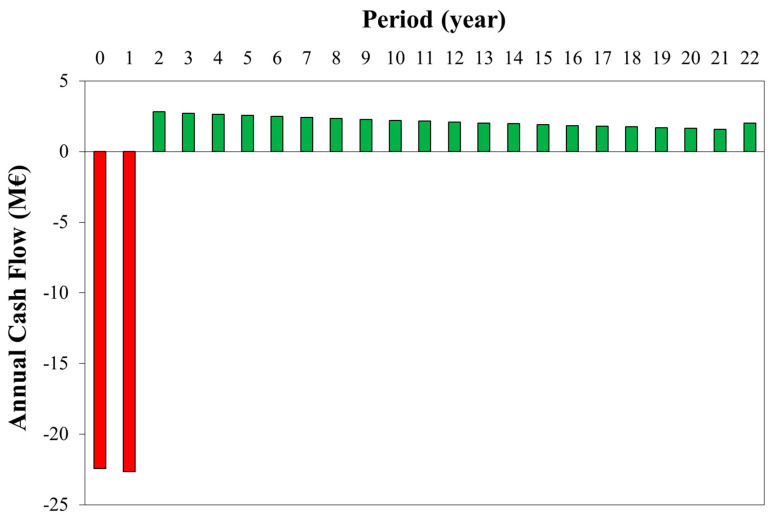
Annual cash flow for the optimal WGS conversion (*x_WGS_* = 40%).

Finally, Figure 8 shows the annual cash flow for the optimal case. After the first two years for the plant construction (about −20 M€), revenues (higher than operating costs) make the annual cash flows positive, and at the 22nd year, the NPV is equal to zero. In the first years, cash flows are higher due to the effect of the discount rate (3%). For the last year, the working capital is recovered, making the cash flow equal to 0.9 M€/y.

Finally, considering the best production cost equal to 0.540 €/kg, it is comparable with the higher end of the cost range for pure hydrogen for Case A considered in Paragraph 2.1 [55]. For a price of pure hydrogen of 6 €/koml_H2_ [32], in fact, 0.449 €/kg is to purchase the pure hydrogen, not considering compression costs and capital costs of the gasifier and compressors.

The obtained results give insights in light of a growing development of a biorefinery concept, and confirm the production of methanol from biomass as a promising strategy to go towards a low-carbon society, although a more detailed study should be carried out [56,57,58,59,60,61,62,63]. For example, the methanol production costs strongly depend on plant capacity. In a recent paper, Wang et al. discussed the effect of feedstock and plant capacity on methanol production costs [64]. In particular, wood, waste, natural gas and coal were considered as raw materials for methanol synthesis. The methanol production cost strongly depends on the plant capacity, especially for the biomass-based processes.

By considering a cost analysis based on 2013 data, for plant capacities from a few tens to hundreds of kton/yr of methanol, the methanol production cost ranges from about 200 to 935 €/ton for either lignocellulose biomass (wood) or waste residues, from 160 to 480 €/ton for coal, and from 90 to 290 €/ton for natural gas. The production of methanol from fossils, especially in the case of natural gas, may be then considered the cheapest solution, although the cost of fossils may change as a function of several factors, such as geopolitics. Therefore, by considering our methanol production rate, namely about 35 kt/yr, our estimation (i.e., 540 €/ton) well agrees with the literature data.

In this work the management of the solid by-product of the gasification, the so-called char, was not considered. From the work of Barisano et al. [21], about 80–100 g of char are produced from 1 kg of dry biomass. If the biomass ash (1.2 wt%) is quantitatively preserved in the produced char, the organic fraction of the char is 88 wt%. The authors analyzed the organic fraction of char: a carbon content of 82 wt%, hydrogen content of about 4 wt% and 14 wt% oxygen [21]. On the basis of these analyses, the solid by-product of the gasification could be considered as biochar [65,66]. Biochar is a versatile product whose applications are continuously expanding, mainly in agriculture, operations related to the natural environment and industry. It can be used as a soil additive, or added to fodder and silage, or applied in water treatment. Biochar can also be used for the immobilization of contaminants from soil, and in sewage treatment; it can be applied as a supplementary material in composting and in methane fermentation processes. Biochar can be used as a catalyst for tar reduction in pyrolysis and gasification, and as a fuel when pelletized [66].

## 4. Conclusions

In this work, a techno-economic assessment of the upgrading of syngas from biomass gasification was studied. In particular, the WGS and CC sections were analyzed, varying the *CO* conversion in the WGS reactor and fixing to the optimal value, an R-ratio (*H*_2_ − *CO*_2_)/(*CO* + *CO*_2_) of 2. The relationship between *CO* conversion and percentage of *CO*_2_ to be removed was studied and the process parameters to obtain the same conditions of the syngas were found. For each case and process condition, an economic analysis was done and set equal to a zero net present value, thus finding the correspondent value of the production cost of methanol. The results show the convenience of lower *CO* conversions with respect to easier *CO*_2_ capturing processes. The main cost parameter is the *H*_2_*O*/*CO* ratio of the WGS section, due to the high natural gas cost. A *CO* conversion equals to 40% was calculated as the optimal value to obtain the lowest process cost with a methanol production cost of 0.540 €/kg.

More detailed studies should be carried out for a more accurate estimation of methanol production cost; but, on the whole, the obtained results reveal that an optimization of the upgrading system should be taken into account.

## Figures and Tables

**Table 1:** The syngas composition considered in this work (average values of the data reported in [21]).

*CO*	*H*_2_	*CO*_2_	*CH*_4_	*N*_2_
30	30	25	10	5

**Table 2:** Process equipment of the considered flowsheet of Figure 2.

Section	Equipment	Equipment Kind	Utility or Steam Required/Produced
WGS	C1	Compressor	Electricity
WGS	E1	Heat exchanger	Natural gas
WGS	E2	Heat exchanger	LPS produced
WGS	P1	Pump	Electricity
WGS	HTS-WGS	Reactor	/
WGS	E3	Heat exchanger	LPS produced
WGS	LTS-WGS	Reactor	/
WGS	E4	Heat exchanger	Cooling water
WGS	F1	Condenser	/
CCS	C2	Compressor	Electricity
CCS	E5	Heat exchanger	Cooling water
CCS	ABS	Absorption column	/
CCS	E8	Heat exchanger	LPS required
CCS	V1	Valve	/
CCS	E6	Heat exchanger	LPS required
CCS	F2	Flash	/
CCS	C3	Compressor	Electricity
CCS	V2	Valve	/
CCS	E7	Heat exchanger	LPS required
CCS	F3	Flash	/
CCS	C5	Compressor	Electricity
CCS	P2	Pump	Electricity
CCS	C4	Compressor	Electricity

**Table 3:** Process variables description.

Section	Process Variable	Units	Range	Effect
WGS	*H*_2_*O*/*CO*	-	1–3	WGS Conversion
WGS	WGS-HTS length	m	0–2	WGS Conversion/ WGS-HTS Pressure drop
WGS	WGS-LTS length	m	0–5	WGS Conversion/ WGS-LTS Pressure drop
CCS	DEPG/GAS	-	0.5–1	*CO*_2_ absorption
CCS	V1 output pressure	bar	10–70	*CO*_2_ absorption/ Captured *CO*_2_ purity
CCS	V2 output pressure	bar	0.7–2	*CO*_2_ absorption/ Captured *CO*_2_ purity

**Table 4:** Economic parameters used in the economic analysis.

Economic Parameter	Units	Calculation	Value	Reference
Biomass supply chain cost (*C_B_*)	€/t		40	[47]
Oxygen purchase cost	€/t		40	[48]
LPS	€/t		7	[49]
MPS	€/t		11	[49]
Natural gas	€/Nm^3^		0.136	[49]
DEPG cost	€/t		5000	[32]
Column packing	€/m3		1000	[32]
Electricity	€/MWh		40	-
Column stage height	M		0.5	[32]
ABS stage	-		12	[32]
Working hour per year	h/y		7920	[32]
F_p_		0.5/7/50 bar	1/1.5/1.9	[49]
F_t_		100/300/500 °C	1/1.6/2.1	[49]
Construction years	Y		2	-
Depreciation years (*y_D_*)	Y		20	[50]
Maintenance and labor (*OC_M_*)	M€/y	% of total capital cost	10	[51]
Working capital (*WC*)	M€	% of total capital cost	2	[32]
Taxes (*t*)	%	of the gross profit	40	[32]
Discount rate (*r*)	%		3	-

**Table 5:** Final variable values.

WGS Conversion (%)	Removed *CO*_2_ (%)	*H*_2_*O*/*CO*	DEPG/GAS	V1/V2 Pressure (bar)
36	98	1	1	25/0.7
40	95	1	1	20/1.6
49	88	2	0.7	21/1.3
59	82	2	0.7	19/1.5
70	76	2	0.5	58/0.7
80	72	2	0.5	28/0.7
90	67	3	0.5	27/1.5
99	64	3	0.5	23/1.1
